# Genetic Diversity and Relatedness among Captive African Painted Dogs in North America

**DOI:** 10.3390/genes12101463

**Published:** 2021-09-22

**Authors:** Cassandra M. Miller-Butterworth, Karen Vacco, Amy L. Russell, Joseph C. Gaspard

**Affiliations:** 1 Penn State Beaver, Monaca, PA 15061, USA; 2 Pittsburgh Zoo & PPG Aquarium, Pittsburgh, PA 15206, USA; kvacco@pittsburghzoo.org (K.V.); jgaspard@pittsburghzoo.org (J.C.G.III); 3 Biology Department, Grand Valley State University, Allendale, MI 49401, USA; russelam@gvsu.edu

**Keywords:** *Lycaon pictus*, African wild dog, Canidae, microsatellite, MHC, DLA-DRB1, D-loop, studbook, relatedness, genetic assessment, ex situ conservation

## Abstract

African painted dogs (*Lycaon pictus,* APD) are highly endangered, with fewer than 7000 remaining in nature. Captive breeding programs can preserve a genetically diverse population and provide a source of individuals for reintroductions. However, most programs are initiated from few founders and suffer from low genetic diversity and inbreeding. The aims of this study were to use molecular markers to assess genetic variation, inbreeding, and relatedness among APDs in the North American captive population, to use these data to realign studbook records, and to compare these data to wild populations and to the European captive population to facilitate the development of a global management plan. We sequenced mitochondrial and major histocompatibility (MHC) class II loci and genotyped 14 microsatellite loci from 109 APDs from 34 institutions in North America. We identified three likely studbook errors and resolved ten cases of uncertain paternity. Overall, microsatellite heterozygosity was higher than reported in Europe, but effective population size estimates were lower. Mitochondrial sequence variation was extremely limited, and there were fewer MHC haplotypes than in Europe or the wild. Although the population did not show evidence of significant inbreeding overall, several individuals shared high relatedness values, which should be incorporated into future breeding programs.

## 1. Introduction

The African painted dog (*Lycaon pictus*), APD, also known as the African wild dog or Cape hunting dog, is a highly endangered canid species facing mounting pressures across its extant populations. The global free-ranging population is estimated at approximately 6600 adults, of which only 1400 are mature, i.e., capable of reproducing during any given breeding season, and their numbers are declining [1]. Due to habitat destruction and fragmentation, infectious disease, and conflict with livestock and game farmers, they have largely been eradicated from north, west, and central Africa, and are likely or confirmed to be extinct in 20 of the 39 countries in which they previously occurred [1,2,3,4,5,6]. A total of 39 subpopulations remain, the largest of which together number fewer than 270 mature individuals [1]. Inbreeding and genetic drift in these small and isolated wild populations has led to loss of allelic richness and heterozygosity over time [7]. Thus, these wild populations remain under threat, and ensuring their sustainability in the long term will require continued legal protection as well as a variety of conservation measures, including translocations and reintroductions [8].

Zoological and wildlife conservation organizations are committed to the preservation of wildlife and their habitats. Several species of amphibians [9], the Arabian oryx [10], California condor [11], European bison [12], Przewalski’s horse [13], and the black footed ferret [14] have been saved from extinction through successful reintroductions of captive animals back into the wild. For such reintroductions to be successful, however, captive populations must be managed to minimize inbreeding, maximize heterozygosity, and accurately represent the genetic diversity of their founder populations in the wild [15]. Genetic management of captive populations is therefore vital for their long-term success. Managed ex situ populations of APD are held in South Africa, Europe, Australia, and North America [16]. These APD in human care may act as a stopgap of extinction through the preservation of genetically diverse populations and as a source of possible reintroductions to bolster dwindling wild populations [17,18].

The North American (NA) population of APD was established in 1955, when the first pair of dogs was imported to the San Diego Zoo [19]. This was followed by regular imports to American Zoological Association (AZA) facilities from a variety of sources, including the private sector, zoos in other regions, and the wild [19]. A studbook for APD [16] is maintained and regularly reviewed to generate recommendations of breeding pairs. Studbooks act as a genealogical guide that begins with founder animals, which, in most cases, were imported from the wild or a facility in the region, and under the assumption that all founders were genetically unrelated. Despite regular imports, the NA breeding population of APD has remained small, and it expanded above 30 individuals only after 1980, peaking at 143 individuals in 2016 [20]. There are currently 136 living APD (67 males:69 females) in the NA population [16]. These individuals are descended from 28 founders, none of which remains in the population [20]. However, studbook records indicate that several founder lineages are disproportionately represented in the population, and the APD Species Survival Plan (SSP), a cooperative effort by member and partner organizations managed under the auspices of AZA and species experts, therefore now prioritizes the breeding of the descendants of founders that have low representation [20]. The AZA aims to maintain the genetic diversity of any managed population above 90% of the founding population. When genetic diversity drops below this benchmark goal, it is expected that reproduction may be negatively impacted by smaller litter sizes, lower birth weights, and increased neonatal mortality [20]. Indeed, high infant mortality (61% for males, 65% for females) has already been documented in the NA population of APD [20], particularly among pups with high kinship [21]. Furthermore, although the maximum observed longevity in captivity is 16 years and 14 years for males and females, respectively, the median life expectancy in NA zoos for dogs that survive the first year of life is only 9.9 years [20]. Highly variable litter sizes, a decline in the number of breeding females due to uterine pathology, and complex pack dynamics pose additional challenges to managing this population [19].

A Population Analysis & Breeding and Transfer Plan (PABTP, [20]) was conducted recently as part of the SSP for APD. In addition to providing breeding recommendations, the PABTP used studbook relationships to estimate current gene diversity (89.84% that of the founders), population mean kinship (MK = 0.1016), mean inbreeding (*f* = 0.0312), and effective population size (*N_e_* = 26.452), and predicted the loss of gene diversity over time [20]. The current gene diversity estimate of 89.84% [20] represented a decline from a previous estimate of 91.3% obtained only two years previously [19]. While the PABTP analyses used modeled projections and demographic analyses of the NA regional studbook data, to date, no molecular genetic analyses have been conducted on the NA population of APD to obtain direct estimates of these genetic measures.

A potential source of error in the PABTP analyses [20] comes from inaccurate and/or missing information in the studbook. While efforts are made to ensure that founder and imported animals are not related to each other and that studbook records are free from errors, this may not always be the case. Background information on imported animals may be limited, uncertain, or unknown, and recording errors can occur [15,18,22,23,24]. Projections of gene diversity, kinship, and inbreeding based only on studbook relationships depend on key assumptions about the founders (i.e., that they were unrelated and non-inbred) and on the studbook being entirely complete and free from error. If these assumptions are incorrect and/or if pedigree information is erroneous or incomplete, this can lead to a significant underestimation of relatedness and incorrect calculations of kinship and inbreeding coefficients [25]. Therefore, as molecular resources and technology have become more readily available and affordable, captive breeding programs are increasingly incorporating genetic evaluations into their management plans. Neutral markers such as microsatellites are most commonly utilized to estimate levels of genetic diversity, inbreeding, and relatedness within a population, as well as to address questions of uncertain parentage or other kinship [18,24,26,27,28,29]. By gaining a better understanding of the genetic diversity and relatedness of the animals housed at zoological institutions, improved recommendations can be made for pairing animals for breeding, the accuracy of studbook information can be clarified, and ultimately, a global management plan to preserve the species can be developed.

The utility of this integrated approach has been demonstrated in the European population of APD. Marsden et al. [18] combined studbook pedigree information with genetic data from ten microsatellite loci as well as the DLA-DRB1 locus of the major histocompatibility complex (MHC), a group of genes involved in immune responses and which may have adaptive importance. The authors compared diversity between captive and wild populations of APD at both the neutral markers and MHC [18]. They found that the European population of APD has retained a large proportion of genetic diversity from its wild founders, likely due to recent imports from South African captive breeding facilities. However, they noted that a substantial proportion of the original founder lineages has been lost due to skewed reproductive contributions from certain founders. They also identified high levels of inbreeding and discrepancies between inbreeding estimates derived from studbook pedigrees versus genetic markers [18].

Although studbooks are utilized in all managed ex situ populations of APD, to date, molecular genetic analyses have been conducted only in the European population. However, effective implementation of a global management plan will require the integration of both genetic data and studbook information from holding institutions in each country. It is also important that similar molecular markers are examined in each population to ensure that the data are directly comparable among populations. The primary aims of this study were (i) to use molecular markers to assess genetic variation, inbreeding, and relatedness among APD in the NA captive population, (ii) to use these genetic data to inform and, where necessary, realign studbook pedigree records, particularly in cases of uncertain or unknown parentage, and (iii) to compare these genetic data to those obtained previously for wild populations [7,18,30,31] and the European captive population of APD [18]. The results from this study will inform future management decisions and ultimately aid in the creation of a global management plan for APD.

## 2. Materials and Methods

### 2.1. Sample Collection

From 2012 to 2017, we sent genetic sampling kits to 37 SSP institutions holding APD in NA, with a request to collect whole blood samples opportunistically during routine veterinary procedures. Samples from 109 APD (61 males: 48 females) were subsequently obtained from 34 institutions, representing 80% of the current population. Blood was collected on FTA Elute collection cards (GE Healthcare, Pittsburgh PA, USA) according to the manufacturer’s instructions, and FTA cards were stored and shipped at room temperature. DNA was extracted from the FTA Elute cards according to the manufacturer’s instructions, eluted into 180 μL 1× Tris–EDTA, and stored at either 4 °C or −20 °C. We included negative controls with DNA extractions to monitor for contamination.

### 2.2. Laboratory Work

To enable comparison with previous work [7,30,31,32], we examined three types of genetic markers used in these previous studies, namely (i) the maternally inherited mitochondrial DNA D-loop control region I; (ii) exon 2 of the DLA-DRB1 major histocompatibility (MHC) class II locus, which has previously been shown to be variable in APD [32]; and (iii) 14 highly variable nuclear microsatellite loci, 10 of which were genotyped in the European APD population [18] and in wild populations [30].

#### 2.2.1. Mitochondrial DNA (mtDNA)

We amplified a 308 base pair (bp) fragment of the mitochondrial D-loop control region I from each sample using canid-specific primers, ThrL and DLH, which were redesigned specifically for APD [33]. Each 25 μL polymerase chain reaction (PCR) cocktail contained 0.4 μM each primer, 5× Q solution (Qiagen, Germantown, MD, USA), ~25 ng DNA, a single GE Healthcare illustra PuReTaq Ready-To-Go PCR Bead (Fisher Scientific, Pittsburgh, PA, USA), and deionized water to volume. Cycling conditions were as follows: 95 °C for 5 min, followed by 30 cycles of 94 °C for 30 s, 55 °C for 30 s, and 72 °C for 30 s, and a final 10 min extension at 72 °C. Amplified products were purified with ExoSAP-IT (Thermofisher Scientific, Pittsburgh, PA, USA). Sanger sequencing was performed in both directions at the Pennsylvania State University Genomics Core Facility, University Park, PA. D-loop sequences were manually edited, trimmed to 308 bp, and aligned by eye using CodonCode Aligner (CodonCode Corporation, Centerville, MA, USA).

#### 2.2.2. Major Histocompatibility Complex (MHC) Locus

Endangered species research has increasingly included assessments of variation at the MHC loci due to the adaptive role that the MHC plays in the immune response to pathogens [34,35,36,37,38,39]. The canid MHC, known as the dog leucocyte antigen (DLA) loci, includes four tightly linked functional nuclear genes that together encode the α and β chains of MHC class II molecules [40]. Previous work on APD has found that two of these genes, DLA-DRA and DLA-DQA1, are monomorphic, and a third, DLA-DQB1, is biallelic [32]. We therefore examined only the polymorphic DLA-DRB1 gene and amplified a 340 bp fragment of exon 2 of this locus in each individual using primers DRBln1 and DRBln2 [40]. Each 25 μL PCR cocktail contained 0.4 μM each primer, 5× Q solution (Qiagen), 5% dimethyl sulphoxide, ~25 ng DNA, one GE Healthcare illustra PuReTaq Ready-To-Go PCR Bead (Fisher Scientific), and deionized water to volume. Cycling conditions, amplicon purification, and sequencing were as described above for mtDNA.

Sequences were manually edited, trimmed to 340 bp, and aligned by eye using CodonCode Aligner (CodonCode Corporation). To facilitate reconstruction of phased haplotypes, we cloned the DLA-DRB1 locus from ten heterozygous individuals. We ligated amplified sequences into pJet1.2/blunt cloning vectors using the CloneJET PCR cloning kit (ThermoFisher Scientific) and subsequently transformed them into DH5α chemically competent cells (ThermoFisher Scientific), according to the manufacturer’s instructions. We randomly selected ten clones from each individual and sequenced them in both directions at the Pennsylvania State University Genomics Core Facility, University Park, PA. Resulting sequences were manually edited and aligned using CodonCode Aligner (CodonCode Corporation).

#### 2.2.3. Microsatellites

To assess neutral genetic diversity, we genotyped each individual at 14 physically unlinked microsatellite loci, which we amplified in five multiplexes: (1) Pez12, FH2054, FH2611, (2) Pez8, FH2010, (3) FH3399, FH3965, (4) FH2785, FH2658, Pez15, and (5) CXX155, CXX250, CXX263, and CXX366 [41,42,43,44,45,46,47]. Amplification conditions have been described in detail previously [48]. Fragment analysis was performed using an Applied Biosystems genetic analyzer (model 3730 XL; Waltham, MA, USA) at the Pennsylvania State University Genomics Core Facility, University Park, PA. One negative control (deionized water) and two previously genotyped samples were included on every plate to monitor for contamination and to ensure consistency across electrophoretic runs.

Allele sizes were called using GeneMarker software (SoftGenetics, State College, PA, USA), based on a known DNA size standard (GeneScan 500 LIZ Dye Size Standard; ThermoFisher Scientific). Each locus was amplified independently two to four times to confirm each genotype, and putative homozygotes were accepted only if the same single allele was observed in at least three independent PCRs. Thereafter, a consensus multilocus genotype was generated for each individual. Consensus microsatellite genotypes were submitted to Dryad (https://doi.org/10.5061/dryad.xksn02vgp).

### 2.3. Statistical Analyses

#### 2.3.1. Mitochondrial DNA (D-Loop) Sequences

We estimated the most appropriate model of nucleotide substitution for the D-loop sequences using jModelTest 2 [49,50]. This was determined to be Jukes–Cantor (JC) with no γ correction. This model was implemented in DNaSP v. 6 [51], which we used to estimate standard measures of genetic diversity, including nucleotide diversity per site (π(JC)), average number of nucleotide differences (*k*), and haplotype (gene) diversity (*H_d_*). Haplotype frequencies were estimated with Arlequin v. 3.5 [52]. D-loop haplotypes identified in the NA population were compared by means of a BLAST search [53] with those previously identified in both wild and captive APD [18,30,31](30: GenBank PopSet 375332022). We used Pedigraph v. 2.4 [54] to construct a pedigree of the sampled dogs, their parents, grandparents, and offspring, based on studbook records. We then manually superimposed onto the pedigree the D-loop haplotype of each sampled individual.

#### 2.3.2. MHC (DLA-DRB1) Sequences

We reconstructed the DLA-DRB1 haplotype pairs most likely to be present in each APD using PHASE v. 2.1 [55,56], with reference to our cloned sequences and to other DLA-DRB1 haplotypes known to occur in *L. pictus* [32]. We identified the most likely pairs of haplotypes present in each APD with >90% probability in all but five dogs. Limited time and funds precluded cloning of this locus in those five individuals and their MHC genotypes remain unresolved. DLA-DRB1 haplotypes identified in the NA population were compared by means of a BLAST search [53] with those previously identified in both wild and captive APD [18,30].

We estimated the most appropriate model of nucleotide substitution for the DLA-DRB1 sequences using jModelTest 2 [49,50]. This was determined to be the Tamura–Nei model with no γ correction. This model was implemented in Arlequin v. 3.5 [52], which we used to generate connections for a minimum spanning network for the DLA-DRB1 haplotypes, which we drew by hand. As for D-loop, we constructed a pedigree using Pedigraph v. 2.4 [54] based on studbook records and manually superimposed onto the pedigree the DLA-DRB1 genotype of each sampled individual. We calculated nucleotide diversity (π), haplotype (gene) diversity (*H_d_*), and the average number of pairwise nucleotide differences per site (*k*) using DnaSP v. 6.12 [51]. We also used DnaSP to test for signatures of selection at this locus. We calculated both Tajima’s D [57], which compares the mean number of pairwise differences and the number of segregating sites under the assumption that they are the same in a neutrally evolving population, and Fu and Li’s D* statistic, which compares the number of singleton mutations to the total number of variants in a genomic region [58]. We estimated observed heterozygosity (*H_O_*) and expected heterozygosity (*H_E_*) in Arlequin v. 3.5 [52], and we used these values to calculate population-level inbreeding, *F_IS_* (*F_IS_* = 1-*H_O_/H_E_*), which is the proportion of genetic variance in a subpopulation that is found within an individual. Negative *F_IS_* values (indicative of an excess of observed heterozygotes) and values close to zero are expected in outbred populations and under random mating, respectively, whereas values close to one suggest inbreeding [59].

#### 2.3.3. Microsatellites

We used MicroChecker [60] and Genepop v. 4.7 [61] to test for the presence of null alleles, linkage disequilibrium, and deviation from Hardy–Weinberg expectations due to either homozygote or heterozygote excess. We used GenAlEx v. 6.5 [62,63] to calculate the probability of identity (*P_(ID)_*, the probability that two unrelated individuals drawn at random from a population will have the same multilocus genotype) and the probability of identity for siblings (*P_(ID)sib_*, the probability of observing identical multilocus genotypes in siblings). We quantified genetic diversity within the NA population of APD by estimating the average number of alleles per locus (*N_A_*) in GenAlEx, and the average number of alleles per locus adjusted for sample size (allelic richness, *R_S_*) using FSTAT [64], as well as average gene diversity over loci, mean observed heterozygosity (*H_O_*), and mean expected heterozygosity (*H_E_*) based on Hardy–Weinberg assumptions using Arlequin v. 3.5 [52]. We also used GenAlEx [63] to estimate population-level inbreeding, *F_IS_ (F_IS_* = 1 − *H_O_/H_E_).*

We estimated pairwise relatedness (coefficient of relatedness, *r*) using both the maximum-likelihood method employed by ML-Relate [65] and the triadic likelihood estimator in COANCESTRY [66], which accounts for inbreeding [67]. The coefficient of relatedness, *r*, is the probability that two individuals share an allele that is identical by descent, i.e., they inherited the same allele from a common ancestor. Unrelated individuals have *r* values close to zero, half-siblings share 25% of their genes (*r* ≈ 0.25), whereas full siblings and parent–offspring dyads share on average 50% of their genes (*r* ≈ 0.5). Values of *r* closer to 1 indicate higher than expected levels of relatedness, suggesting that those individuals are inbred, i.e., their parents were closely related. We estimated the most likely relationships between individuals (unrelated, half-siblings, full-siblings, parent–offspring) using ML-Relate, and implemented 100,000 randomizations to estimate 95% confidence intervals for putative relationships. We then compared both *r*-values and putative relationships between pairs of individuals identified by ML-Relate with those listed in the studbook. ML-Relate does not identify grandparental or avuncular relationships (*r* ≈ 0.25), or first cousins (*r* ≈ 0.125). Instead, these would likely be categorized as half-siblings or unrelated based on their *r*-values. We therefore considered these two ML-Relate categories to be consistent with studbook records for individuals that were first cousins or that shared a grandparental or avuncular relationship. We compared pairwise relatedness (estimated by ML-Relate) among relationship categories recorded by the studbook (parent–offspring, full siblings, half-siblings, avuncular, and first cousins) using a one-way ANOVA and a post hoc Tukey honestly significant difference (HSD) test.

We estimated the contemporary effective population size, *N*_e_, of the NA population of APD using the single-sample bias-corrected method based on linkage disequilibrium [68], as implemented in NeEstimator v. 2.1 [69]. Analyses were run with the full dataset of 109 individuals and 14 microsatellite loci. Due to missing data (at least one allele missing for ≤32 individuals per locus), we excluded alleles with frequencies lower than 0.006. Wild populations of APD have a hierarchical mating system dominated by a single breeding pair [70], but efforts have been made to minimize inbreeding in captivity. Therefore, we estimated *N*_e_ both under the assumption of monogamous and, separately, random mating. Error in this estimate was quantified as a 95% confidence interval using a nonparametric jackknife approach [71]. As a point of comparison, we also estimated *N*_e_ for the European APD population using the same method. Microsatellite data for the European population came from Marsden et al. [18] and constituted 214 individuals genotyped at nine loci. For this analysis, we used the settings described previously, but excluded alleles with frequencies lower than 0.002.

Studbook records were obtained for each sampled individual. For 43 APD, DNA was available from the individual as well as from both presumptive parents, and we were able to test the probability of maternity, paternity, and the family trio using Cervus 3.0 [72]. This software implements a likelihood analysis of candidate parents based on Mendelian inheritance of microsatellite genotypes of the individuals concerned, and the relative frequencies of alleles at each locus [72]. In 11 cases, paternity was listed in the studbook as uncertain and could be assigned to either of two full brothers. We used Cervus to test the probability of paternity of both potential sires, given the known dam. For five additional APD, DNA was available from the sire only, and for a further 18 dogs, DNA was available from the dam only. In these samples, we used Cervus to test the likelihood of either paternity or maternity -, given that the second parent was unknown. We used both the LOD score (the natural log of the overall likelihood ratio) and Delta values to assess the relative likelihoods of candidate parents. A candidate parent with a positive LOD score is more likely to be the true parent than to not be the true parent, whereas the reverse is true of a candidate parent with a negative LOD score [72]. If two or more candidate parents have positive LOD scores, a derivate of the LOD score, the Delta value, can be used to assign parentage. Delta is the difference in LOD scores between the most likely and the second most likely candidate parents [72]. Where possible, inheritance of DLA-DRB1 haplotypes was also used to support or exclude candidate parents.

## 3. Results

### 3.1. Mitochondrial DNA (D-Loop)

The D-loop sequences had low nucleotide diversity (π(JC) = 0.00483 ± 0.00016 SD) and haplotype (gene) diversity (*H_d_* = 0.491 ± 0.017 SD). The average number of nucleotide differences, *k*, was 1.473. There were only three polymorphic sites, all transitions, in the 308 bp fragment, resulting in only two haplotypes, both of which have previously been identified in APD [30,31]. Haplotype S2 was present in 65 individuals (frequency = 0.596) and haplotype Z1 was present in 44 individuals (frequency = 0.404; Supplementary Figure S1; GenBank accession numbers MZ825436-MZ825437).

### 3.2. MHC (DLA-DRB1)

**Figure 1:**
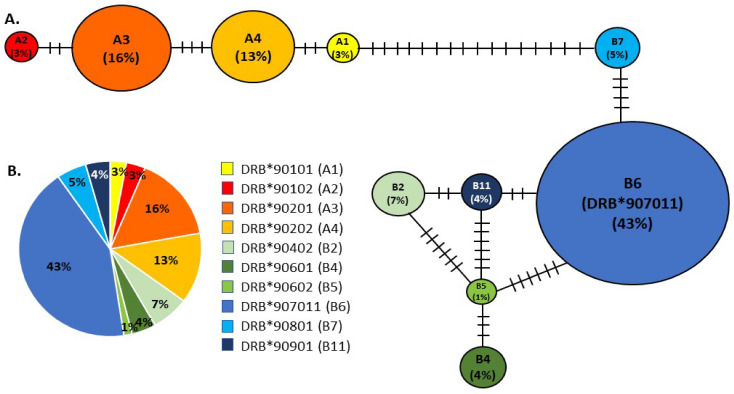
**(A**) Minimum spanning network of MHC DLA-DRB1 haplotypes identified in sampled APD from the NA population. Sizes of the circles represent the frequency of the haplotype in the population; hatch marks represent the number of mutational events separating haplotypes. (**B**) Pie chart illustrating the relative frequencies of each DLA-DRB1 haplotype in the NA population.

We identified ten DLA-DRB1 haplotypes, all with unique amino acid sequences (Figure 1), although none was unique to the NA population (Table 1; GenBank accession numbers: MZ825438-MZ825447). BLAST [53] searches matched all haplotypes with 100% identity to APD nucleotide and amino acid sequences for DLA-DRB1. Furthermore, there was no evidence of more than two sequences per individual or of pseudogenes (no frameshift mutations or stop codons were identified), indicating that we had amplified a single functional DLA-DRB1 locus. DLA-DRB1 haplotype pairs were reconstructed with >90% probability for 104 APD using experimental or computational phasing. Eighty-eight of these 104 individuals (84.6%) were heterozygous. For 28 of these heterozygous individuals, DNA was also available from their presumptive parents (as listed in the studbook), and we were able to infer which haplotype was inherited from the dam or from the sire.

The DLA-DRB1 locus was more variable than the D-loop as there were 30 polymorphic sites in the 340 bp fragment (9 transitions, 21 transversions). In the translated amino acid sequence, there were 27 segregating sites. Nucleotide diversity, π, was 0.036 ± 0.001, the average number of pairwise nucleotide differences, *k*, was 11.359, and haplotype (gene) diversity, *H_d_*, was 0.796 ± 0.025. Both observed (*H_O_*) and expected heterozygosity (*H_E_*) were moderately high (*H_O_* = 0.836; *H_E_* = 0.7653 ± 0.024) and were not significantly different from each other (*p* = 0.279). The fixation index (*F_IS_* = 1 − *H_O_/H_E_*) was −0.093. Significantly positive values for both Tajima’s *D* (*D* = 3.499, *p* < 0.001) and Fu and Li’s D* (D* = 2.009, *p* < 0.02) suggested that this region may be under balancing selection [73].

**Figure 2:**
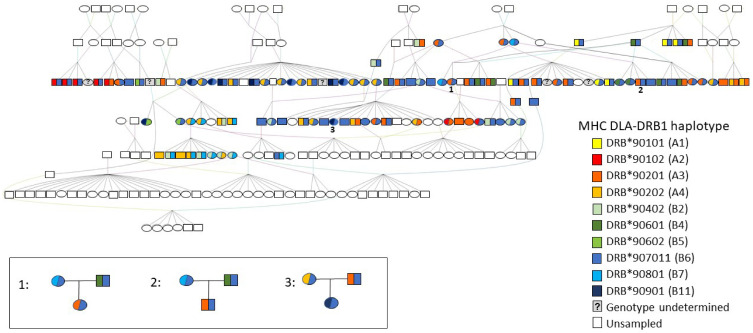
Pedigree of a subset of the NA population of APD, including sampled individuals (*n =* 109), their parents, grandparents, and offspring. Squares represent males; ovals represent females. Colors indicate MHC DLA-DRB1 genotypes of sampled individuals; individuals with a single color are homozygous for the represented allele; those with two colors are heterozygous. Three cases are highlighted in the inset (numbered 1–3 on the pedigree), where the MHC genotypes of the offspring are inconsistent with those of the presumptive parents, indicating likely errors in the studbook.

The DLA-DRB1 haplotypes formed two divergent allele lineages (A and B) in the minimum spanning network (Figure 1). Haplotypes within each lineage differed by 2–6 mutational changes, whereas the minimum difference between haplotypes from different lineages (Haplotypes A1 and B7) was 16 mutational changes. Haplotype B6 (also referred to as allele DRB*907011) was the most common (frequency 42.8%, Figure 1). It was detected in 77 (74%) individuals, 12 of which were homozygous, and it was distributed across most family groups. This is illustrated in the pedigree (Figure 2) of dogs included in this study, together with their ancestors and descendants. Other haplotypes ranged in frequency from 1.44% (B5 or DRB*90602, detected in 3 (2.9%) individuals) to 15.87% (A3 or DRB*90201, detected in 33 (31.7%) individuals, three of which were homozygous; Table 1, Figure 1 and Figure 2).

### 3.3. Microsatellites

#### 3.3.1. Genetic Diversity

A complete 14-locus genotype was obtained for 91 individuals (83.5%). A further 16 individuals (14.7%) were genotyped at 13 loci, and 2 individuals (1.8%) were genotyped at 12 loci. The probability of identity (13 loci *P_(ID)_* = 1.9 × 10^−14^; 14 loci *P_(ID)_* = 3.5 × 10^−15^) and the probability of misidentifying siblings as the same individual (13 loci *P_(ID)sib_* = 6.2 × 10^−6^; 14 loci *P_(ID)sib_* = 2.9 × 10^−6^) were extremely low. The average allelic richness (*R_S_*) for all individuals (*n =* 109) sampled was 6.366 ± 2.598 SD (Table 2). Mean unbiased expected (*H_E_*) and observed (*H_O_*) heterozygosities across all loci were both moderately high (*H*_E_ = 0.746 ± 0.086 SD, *H_O_* = 0.816 ± 0.091 SD). The mean overall coefficient of inbreeding (*F_IS_*) was −0.108 ± 0.141 SD. Similar analyses limited to the same 10 loci used by Marsden et al. [18,30,74] were not significantly different from those of the full dataset of 14 loci (unpaired *t*-test, *p* ≥ 0.147); thus, we present analyses of the full dataset. None of the loci showed significant evidence of null alleles, but several pairs of loci displayed significant linkage disequilibrium (LD). Multiple loci also deviated from Hardy–Weinberg equilibrium (HWE) expectations due to an excess of heterozygotes. However, these results are not unexpected because, while HWE and LD analyses assume independent lineages, this captive population consists of closely related family lines that, by definition, violate the assumptions of HWE and of an LD model. Furthermore, heterozygote excess can be indicative of a population bottleneck [75], which is expected in a small, captive population. Statistical LD between pairs of microsatellite loci was also reported in studies of wild APD packs and was attributed to oversampling of fecal samples from related individuals within each pack [7,31]. These authors also did not exclude the affected loci from their subsequent analyses.

#### 3.3.2. Relatedness

**Figure 3:**
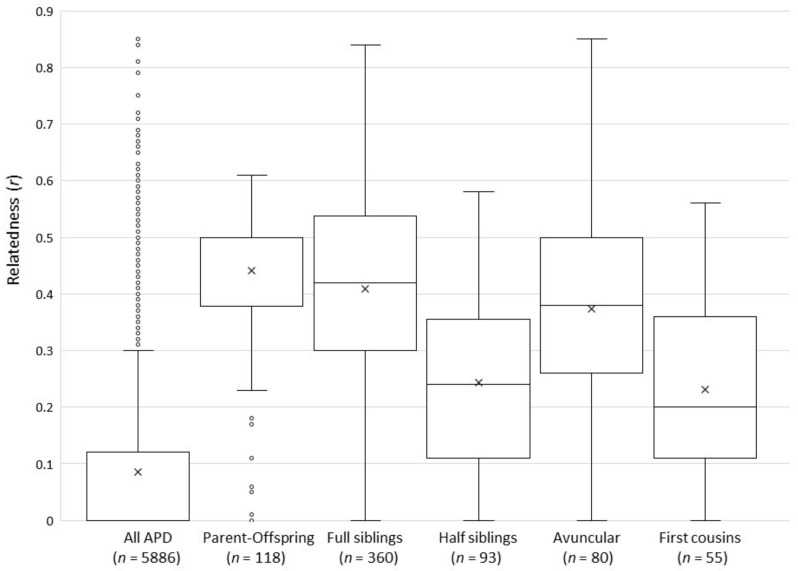
Box and whisker plots of pairwise relatedness coefficients (*r*) between pairs of APD, including the entire NA sample and five categories of relationships as defined by studbook records. Mean relatedness is indicated by an X within each box; median is indicated by a horizontal line within each box; outliers are shown by open circles above or below whiskers. The numbers of pairwise comparisons conducted within each defined relationship category are indicated in parentheses (*n*).

ML-Relate and Coancestry produced similar pairwise relatedness estimates. The overall Pearson’s correlation coefficient between methods was 0.959; therefore, only ML-Relate results are reported here. Average relatedness between all sampled APD (*n =* 109) was low (mean *r* = 0.086 ± 0.146 SD, range *r* = 0–0.85, Figure 3), and only 49 of 5886 (0.83%) possible pairwise relationships had higher than expected relationship coefficients (*r* > 0.6). However, these included ten pairs of individuals that had relatedness values between 0.713 and 0.850, suggesting that their parents were closely related. Nine of these pairs were classified as full siblings in the studbook, and one pair shared an avuncular (uncle–niece) relationship. As described in the Methods, we considered ML-Relate categories of half-siblings and unrelated to be consistent with studbook records of first cousin, grandparental, or avuncular relationships. Allowing for these ambiguities, 96.4% of the relationships (5675 of 5886 possible pairwise comparisons) identified by ML-Relate were consistent with studbook records. The 3.6% that were inconsistent with studbook relationships were generally misclassified by ML-Relate due to unexpectedly high or low relatedness values between pairs of individuals. Overall, based on relationships identified by the studbook, mean relatedness (Figure 3) between parents and offspring (*r* = 0.440 ± 0.127 SD; range: 0–0.610), between full siblings (*r* = 0.409 ± 0.166; range 0–0.840), and between half-siblings (*r* = 0.246 ± 0.161, range 0–0.580) was similar to expected levels for each relationship category; however, the range of relatedness values was broad in each case. In contrast, mean relatedness between dogs sharing avuncular relationships (*r* = 0.373 ± 0.154; range 0–0.850) was higher than expected. Although avuncular relationships are expected to have relatedness values similar to those of half-siblings (approximately *r* = 0.25), relatedness between avuncular dogs in the APD was significantly higher than those identified as half-siblings in the studbook (one-way ANOVA post hoc Tukey HSD Q = 7.84, *p* < 0.00001), but their relatedness did not differ from those listed as full siblings (one-way ANOVA post hoc Tukey HSD Q = 2.09, *p* = 0.578). Furthermore, first cousins are expected to have low relatedness values of approximately *r* = 0.125, but relatedness between dogs identified in the studbook as first cousins was higher than expected (*r* = 0.231 ± 0.160; range 0–0.560) and did not differ from that between half-siblings (one-way ANOVA post hoc Tukey HSD Q = 0.72, *p* = 0.987). These higher than expected relatedness values could be due to inbreeding, or errors in studbook records, or a combination of both.

#### 3.3.3. Parentage

In the majority of the 43 cases where it was possible to test both the maternity and paternity of individuals, the most likely trios of parents and offspring were consistent with studbook records. However, in eight cases, an alternative dam, and in four cases, an alternative sire, was identified with similar likelihood scores to the parent listed in the studbook and could not be excluded as the true parent based on microsatellite genotypes alone. In two of these cases, where two potential dams (full sisters) had similar, positive LOD scores and Delta values close to zero (meaning that neither could be excluded as the true mother), DLA-DRB1 haplotypes of both candidate mothers, the offspring, and the sire were known and were used to exclude one putative dam. The non-excluded candidate dam was consistent with the studbook record in both cases.

For a further four individuals, the most likely dam and/or sire identified with Cervus had higher LOD scores than the parent listed in the studbook; this result, in combination with high Delta values, indicated that they were much more likely to be the true parent than the individual listed in the studbook. Furthermore, ML-Relate estimated lower relatedness (*r* = 0–0.31) between the offspring and the parent listed in the studbook, and between the offspring and putative full siblings, than between the offspring and the alternative candidate parent (*r* = 0.35–0.54). Additionally, the combinations of dam and sire listed in the studbook were not possible based on the inheritance of DLA-DRB1 haplotypes identified in the offspring (Figure 2), but these haplotypes were consistent with the alternative candidate parents identified by Cervus. In three of these cases, the Species360 Zoological Information Management Software (ZIMS) database, which zoological institutions use to maintain individual animal records, indicated that the alternative dam identified by Cervus was in fact the correct mother and that the studbook records for these individuals were erroneous. In the fourth case, the ZIMS database and the studbook records of dam and sire were consistent with each other, but Cervus, ML-Relate, and MHC haplotype analyses indicated that the listed sire was most likely incorrect. We were not able to identify the correct sire in this case and suggest that the true father was not included in our sample.

Using inheritance of both microsatellite alleles and DLA-DRB1 haplotypes, we were able to resolve ten of the 11 cases of uncertain paternity where the putative sires were full brothers. In only one case, neither of the two brothers could be excluded as the potential sire as both had similar, positive LOD scores, and DLA-DRB1 haplotypes were uninformative. However, it should be noted that these parentage results represent statistical likelihoods that are based exclusively on the samples available. It is possible that another individual who was not sampled could be the true parent in each case.

#### 3.3.4. Effective Population Size (N_e_)

We estimated the contemporary effective size of the NA population of APD as 17.7 (95% CI 14.7–21.3), assuming that the population is managed in a way that approximates random mating (Table 3). This estimate increases as mating becomes less random, up to 37.1 individuals (95% CI 31.2–44.1) if the population is managed to approximate a monogamous mating system. This tells us that the actual NA population of APD with a census size of 137 individuals [16] is evolving and losing alleles due to genetic drift at the same rate as an ideal Wright–Fisher population of 17.7 to 37.1 individuals. The effective size of the European APD population was estimated to be slightly larger (random mating: *N*_e_ = 23.9, 95% CI 20.1–28.3; monogamous mating: *N*_e_ = 49.5, 95% CI 42.0–58.2) than the NA population. Although the census size of the European population according to Species360 ZIMS (*n* = 247) is nearly twice that of the NA population, the estimated effective size of the European population was only 1.33–1.35× larger than that of the NA population, depending on the mating system assumed.

## 4. Discussion

Listed as endangered by the International Union for Conservation of Nature [1], APD populations have declined dramatically over the past 50 years and have disappeared from much of their former range. Ex situ conservation efforts, including captive breeding programs, therefore play an increasingly important role in preventing the extinction of this iconic species. The first internationally recorded APD in captivity was at the New York Bronx Zoo in 1902, and the first internationally recorded birth in captivity was at the same zoo in 1942 [16]. As this species survives well and successfully rears offspring in captivity [16], the global captive population has since expanded to >700 individuals, which includes established captive breeding programs in North America, Europe, Australasia, and South Africa. Through careful management and the use of studbooks, each region aims to maintain a viable, genetically healthy population by coordinating breeding efforts across multiple institutions. Ultimately, the goal of these programs is to develop a global management strategy for the species that can buffer wild populations, conduct research, raise awareness of, and focus attention on the problems facing wild populations, and potentially provide a source of animals for reintroductions into the wild [16]. The NA captive breeding program (managed by the AZA) forms a critical component of these ex situ conservation efforts. With 137 living APD [16], it holds nearly 20% of the global population under human care, and it is therefore essential that it minimizes inbreeding and maintains high levels of genetic diversity in this population.

The PABTP [20] estimated gene diversity, inbreeding, and kinship through modeled projections and demographic analyses of the NA regional studbook data. This document predicted that the population would lose an additional 10% of its gene diversity within 20 years and would retain only 51.1% by 100 years [20]. As a result, APD qualify as a Yellow SSP, a designation given to AZA species that have a population size greater than 50 but which cannot maintain 90% gene diversity for 100 years or 10 generations. However, molecular genetic analyses of the NA population of APD have been lacking. Here, we used both mitochondrial and nuclear genetic markers to assess the genetic health of the NA population of APD, to inform and, where necessary, update studbook records, and to validate previous demographic estimates of gene diversity, kinship, and inbreeding. Furthermore, our data enabled us to compare the genetic diversity of the NA population of APD to those in Europe and in the wild.

### 4.1. Comparison of mtDNA Haplotypes with Managed European and with Wild Populations

Although several studies have examined the genetic diversity of APD in wild and managed populations in southern africa and east africa [7,8,30,31,32,74,76], until now, only one other comprehensive molecular genetic analysis of APD in captivity has been conducted. Marsden et al. [18] examined the same molecular markers (D-loop, DLA-DRB1, and microsatellites) as our study in the European captive APD population and compared them to wild and captive populations in South Africa. They did not specifically report D-loop measures of nucleotide or haplotype (gene) diversity for the European population of APD; therefore, it is not possible to compare these measures of sequence diversity directly between the NA and European populations. However, D-loop nucleotide diversity in the NA dogs was higher than that reported in wild populations of APD in South Africa, which ranged from π = 0 to 0.0018 in the Lowveld and Kruger National Park regions [7,30]. Slightly higher D-loop nucleotide diversity has been found in populations in Zimbabwe (π = 0.0004–0.0119; [7,30]), with the highest levels being recorded in the Okavango Delta, Botswana (π = 0.0222; [74]), and east Africa (π = 0.0062–0.01942; [74]).

Marsden et al. [18] identified three D-loop haplotypes (S2, S5, and Z1) in the European population, whereas we detected only two in the NA population, S2 and Z1 (Supplementary Figure S1). However, the haplotypes were more equally represented in the NA population than in Europe, which was dominated by haplotype S2 (frequency 75%; [18]). In contrast, up to ten D-loop haplotypes have been identified in wild populations of APD. The prevalence of both haplotypes S2 and Z1 in the NA population is consistent with the female founders of this captive population having southern African rather than east African origins. Neither haplotype S2 nor Z1 has been detected in east African populations of APD [30,31], but several studies have reported frequencies of S2 in southern Africa, ranging from 14% in the Okavango Delta, Botswana to between 60 and 100% of individuals sampled from Kruger National Park, South Africa, and 100% of the dogs sampled from Namibia [7,18,30,31]. Haplotype S2 was also found at 60–100% frequency in all captive populations examined in South Africa and Zimbabwe [18,31]. In contrast, the Z1 haplotype appears to be less common in wild southern African populations. Girman et al. [31] identified Z1 in only one individual from the Okavango Delta, Botswana, and one from Hwange in Zimbabwe, although these authors did record both S2 and Z1 in museum skins originating from Malawi, Botswana, Zimbabwe, and South Africa. Similarly, Marsden et al. [18,30] identified Z1 only in Okavango, Botswana (4% frequency), and Hwange, Zimbabwe (23%), and Tensen et al. [7] found Z1 in Zimbabwe but not in South Africa.

The high frequency of the Z1 haplotype in the NA population (~41%) is likely the consequence of a founder effect compounded by subsequent unequal founder contributions to subsequent generations. According to the AZA studbook [16], all the dogs included in this study are descended from six wild-caught female founders captured in South Africa, Namibia, and Botswana. All sampled individuals having haplotype Z1 (*n =* 44) are descended from two female founders captured in 1998 in Botswana [16], where Z1 is more common [30,31], but 43 of these individuals are descended from one of these founders, the other having had only two male descendants (Supplementary Figure S1). Of the 65 sampled individuals with the S1 haplotype, 88% (*n* = 57) are descended from two females from South Africa, and 12% (*n* = 8) are descended from two females from Namibia [16]. Reproductive skew has thus led to higher frequencies of the Z1 haplotype in the NA population than is found in the wild, together with the loss of other haplotypes such as S5, which is present in Europe, and potentially others (S1, S5, Z2, E1, and E2) that are found in wild southern African populations [18,31] but were not identified in the NA population. Such over-representation of certain individuals and the subsequent loss of genetic diversity is not uncommon in captive breeding programs. It has similarly been recorded in the European population of APD [18], as well as in other species [28,77,78].

### 4.2. Comparison of MHC Haplotypes with Managed European and with Wild Populations

Balancing selection at the DLA-DRB1 locus appears to have resulted in more polymorphic sites and higher nucleotide and haplotype diversity than at the noncoding mtDNA D-loop sequences. Although nucleotide diversity was lower than that reported for wild populations in southern Africa and east Africa, which ranged from π = 0.051 to 0.076 [32], observed and expected heterozygosities for the NA population were moderately high and were comparable to wild populations (southern Africa range *H_O_* = 0.67–0.84, *H_E_* = 0.66–0.86; east Africa range *H_O_* = 0.54–0.91, *H_E_* = 0.57–0.88), and to Europe (*H_O_* = 0.83; *H_E_* = 0.84) [18,30,32]. The weakly negative fixation index was also similar to that reported by Marsden et al. [18,30] for Europe (*F_IS_* = 0.01) and for wild populations (*F_IS_* = −0.08), and suggests an outbred population [59].

Previous work on APD [32] has shown that, while this species had a comparable number of alleles or haplotypes at the DLA-DRB1 locus (17 in total), overall amino acid variation was lower than that of other canids. Our findings of low MHC diversity are consistent with these previous reports. We identified ten different DLA-DRB1 haplotypes (Table 1, Figure 1) in the NA population, which formed two highly divergent lineages, A and B. All ten of these haplotypes, and lineages A and B, have been reported previously in wild populations of APD from southern Africa or east Africa [30,32] and in Europe [18]. However, seven haplotypes previously identified in wild populations were lacking in the NA dogs, including three (A7, B1, and B13) that were found in Europe [18]. One haplotype (B4) was found in NA but not in Europe, although it was reported from wild populations in both east Africa and southern Africa [18,32]. The NA population was dominated by haplotype B6 (frequency 43%; Table 1), which was present in 77 individuals, 12 of which were homozygous (Figure 2). Haplotype B6 was also most common in wild southern African populations (frequency 22.5% [30]), particularly in the Lowveld and Kruger National Park regions of South Africa (frequency 54% [30]). In contrast, the frequency of haplotype B6 in the European population was only 5.2%, and this population was instead dominated by haplotype A1 (frequency 32.2% [18]), which was rare in NA (frequency 2.9%). According to the AZA studbook [16], the majority of the 28 wild-caught founders were captured in South Africa (*n =* 15, 54%) and Namibia (*n =* 8, 29%), two were caught in Botswana, and three have unknown origins. The high frequency of the DLA-DRB1 haplotype B6 in the NA population, as well as the presence of haplotypes A2 and B11, which have not been found in east Africa [30], are consistent with a southern African origin for the population founders and suggest that the three founders with unknown origins likely also came from southern Africa.

### 4.3. Comparing Microsatellite Diversity and N_e_ with Managed European and with Wild Populations

Both expected and observed heterozygosities at microsatellite loci were lower than the gene diversity (89.84%) estimated from the studbook for the PABTP [20]. However, microsatellite variation in the NA population was generally higher than that reported for Europe and for wild populations of APD (Table 2). Estimates of expected heterozygosity were similar between captive programs (European *H_E_* = 0.73), but observed heterozygosity (European *H_O_* = 0.76) and allelic richness (European *R_S_* = 4.8) were both higher in the NA than in the European population [18]. The NA population has also maintained higher microsatellite diversity than captive colonies in southern Africa (*H_E_* = 0.504 [31]), as well as wild southern African and east African populations. Girman et al. [31] examined wild populations ranging from South Africa to Kenya and found low numbers of alleles per locus (*N_A_* = 3.4–4.4) and low expected heterozygosity (*H_E_* = 0.504–0.655). Tensen et al. [7] reported very low allelic richness (*R_S_* = 2.66–3.06) and expected heterozygosity (*H_E_* = 0.514–0.577) in the Lowveld region of South Africa, although they found higher genetic diversity in the nearby Kruger National Park (*R_S_* = 3.79; *H_E_* = 0.706). In contrast, Marsden et al.’s [30,74] estimates of genetic diversity were higher for all wild populations that they examined, ranging from the Masai-Steppe in Kenya (*R_S_* = 4.31; *H_E_* = 0.61; *H_O_* = 0.62) to Hwange in Zimbabwe (*R_S_* = 5.68; *H_E_* = 0.76; *H_O_* = 0.80). Tensen et al. [7] noted that the genetic diversity of APD in the Lowveld and Kruger National Park in South Africa had declined in the 5–15 years (1–3 generations) between sample collection for their study and that of Marsden et al. [30], which included samples from the same region. They proposed that inbreeding and genetic drift due to reduced gene flow likely led to a reduction in *R_S_* and *H_E_* in these populations, which was supported by high private allelic richness in Kruger National Park [7]. Although Girman et al. [31] examined different microsatellite loci to those used here, 10 of the loci used in the other studies of wild and captive populations [18,30,74] are the same as in our study, and so our estimates of genetic diversity are directly comparable and suggest that microsatellite diversity in the NA population is higher than that in Europe and several wild populations of APD. This is further supported by analyses (not shown) indicating that diversity estimates from a subset of the NA dataset using just the 10 loci examined in other studies [18,30,74] are not significantly different from the reported estimates derived from the complete NA dataset.

Microsatellite genotypes have also been used to estimate contemporary effective population size (*N*_e_) in captive and wild populations of APD using the linkage disequilibrium method of Waples and Do [68]. Our analyses show that, between the European and NA captive populations, the estimated *N*_e_ of the European population (based on data from Marsden et al. [18]) is slightly larger than for NA, although the confidence intervals overlap (Table 3). We further found that the assumed mating system has a large influence on the estimated *N*_e_ and encourage those using this analysis to specify this setting when reporting results. It is difficult to summarize the true “mating system” of APD in captivity; the animals are monogamous in nature [70] but are managed in captivity, with the intention of minimizing inbreeding and maximizing genetic diversity. Thus, we recommend that our estimates of *N*_e_ under random and monogamous mating be considered a minimum and maximum of reasonable values for each captive population. *N*_e_ estimates from wild populations in southern Africa using the same LD-based method tend to be higher than our estimates from captivity, ranging from 21.8 to 79.1, with higher estimates from Zimbabwe than from South Africa [7]. On the other hand, *N*_e_ estimates from wild populations from east Africa are similar to or even lower than those from captive populations (*N*_e_ = 3.0–21.5; [74]). We note that reports of these *N*_e_ estimates from wild populations do not specify the assumed mating system; monogamy is likely the better approximation for wild populations, but random mating is the default setting for the NeEstimator software. If the default settings were used for these analyses of wild populations, the values reported are likely underestimates.

A primary goal of captive breeding programs is to maximize the preservation of the genetic diversity of the species as a whole, which requires that founders be sourced from a variety of populations and that the captive population is not dominated by a minority of genetic lineages from a single region [15]. Furthermore, it is important to define key management units of wild populations and for breeding programs to focus on threatened lineages [15]. Despite the loss of many founder mtDNA and MHC lineages, the high diversity metrics for microsatellites compared to the European and wild populations suggest that the NA population of APD has maintained good representation of the genetic diversity of the southern African populations from which this captive breeding program was derived. However, this is the only region that is represented in NA. The most highly threatened wild populations of APD, located in east, central, and west Africa, are not represented in NA or in Europe [18]. Wild APD populations are strongly substructured, with minimal gene flow between them [7,8,30,31], and so could be considered separate management units. However, the current global captive breeding program for APD is not representative of the genetic diversity of the species as a whole, and with its heavy bias towards southern African diversity, the NA dogs would potentially be suitable only for reintroduction programs in southern Africa [18].

### 4.4. Inbreeding, Relatedness, and Parentage Queries

Overall levels of relatedness (Figure 3) and inbreeding at microsatellite loci in the NA population were low and similar to inbreeding estimates reported for Europe (*F_IS_* = −0.05) and for wild populations (*F_IS_* = −0.06) [18]. Mean relatedness and the negative *F_IS_* are also consistent with the low mean kinship (MK = 0.1016) and inbreeding coefficient (*f =* 0.0312) estimated for the PABTP [20], although these pedigree-based values are estimated differently and so are not directly equivalent to the genetic-based values [15]. Nevertheless, a correlation between the inbreeding values has been reported in other studies of captive breeding programs [25].

Although relatedness and inbreeding in the population as a whole were low, we found that multiple pairs of individuals had much higher than expected *r*-values. In some cases, this could be due to erroneous parentage assignments (discussed below). We identified three cases in the studbook in which individuals assumed to be half-siblings were in fact full siblings, or individuals recorded as having an avuncular relationship were in fact parent and offspring. This could explain some pairs of half-siblings that shared *r*-values up to 0.58 (Figure 3), which is more typical of full siblings. However, pedigree errors cannot explain all the high *r*-values, particularly the very high relatedness between an uncle and niece (*r* = 0.850) and between nine pairs of full siblings (*r* = 0.713–0.840), suggesting that these individuals are inbred, i.e., share parents that are closely related. The studbook [16] confirmed that the parents of two such full sibling pairs (*r* = 0.75 and 0.79) were full siblings themselves. Furthermore, the mean relatedness (Figure 3) of individuals identified in the pedigree as first cousins and as having avuncular relationships was twice as high as expected for these types of relationships and did not differ from relatedness between half-siblings (r ≈ 0.25) and full siblings (r ≈ 0.5), respectively. It is therefore likely that multiple individuals within these categories are inbred. This should be taken into consideration when planning future breeding recommendations.

Over 90% of the parent–offspring trios predicted by Cervus, and over 96% of the pairwise relationships predicted by ML-Relate, were consistent with studbook records for those individuals, confirming the full sibships and parent–offspring relationships recorded in the pedigree. Furthermore, we were able to resolve with high confidence 10 of 11 documented cases of uncertain paternity. These occurred in two families, and, in both cases, we found that only one of the two possible males sired all the offspring within that family. However, we also identified several likely errors in the studbook records for both dams and sires, which have implications for future breeding and transfer planning, particularly as such errors may lead to underestimation of relatedness and inbreeding in the population inferred in the absence of genetic data. This problem is not unique to the AZA studbook, with similar discrepancies having been documented in the APD studbook in Europe [18], as well as in studbooks of other species [22,23,24,77,79,80,81]. In the NA population, we identified four APD whose parentage (three dams, one sire) was inconsistent with studbook records. In three of these cases, reference to the 360Species ZIMS database confirmed an error in the studbook and indicated that the true dam was the sister of the female recorded in the studbook, thereby validating our prediction with Cervus. In the fourth case, all molecular genetic analyses indicated that the sire listed in both 360Species ZIMS and the studbook could not be the true sire, and both databases therefore likely contain an error; however, we were not able to identify the true father of this female in our sample. Although she shared low *r*-values with individuals identified in the studbook as being her full siblings (*r* = 0–0.32), she shared very high *r*-values with presumptive aunts and uncles (*r* = 0.56–0.85), suggesting that her parents were closely related (i.e., she was inbred). Furthermore, this female was heterozygous B6/B11 at the DLA-DRB1 locus, and the B11 haplotype was found in only 4% of sampled individuals (colored red in Figure 1). With the exception of this female, all individuals having the B11 haplotype were her dam’s siblings and one of their offspring. It is possible, therefore, that one of these presumptive uncles is her true sire, rather than the male listed in the studbook. If so, this would be consistent with our predictions from both ML-Relate and Cervus.

## 5. Conclusions

In conclusion, our analyses demonstrate the important role of direct genetic information in managing captive populations, with the goal of maximizing genetic diversity, minimizing inbreeding, and supplying individuals for reintroduction into the wild. Our analyses confirmed the majority of relationships described in the AZA studbook, but documented significant skew in the reproductive contribution of founders, leading to high levels of inbreeding in some lineages and loss of founder diversity, particularly at D-loop and MHC loci. We were also able to identify some errors in the studbook and clarify most instances of uncertain parentage, and we recommend that updates be made to both the studbook and 360Species ZIMS database in accordance with our findings. Remaining uncertainty stems mainly from a lack of full sampling of the NA population of APD; we encourage full participation of NA institutions housing APD in genetic analyses as a means of achieving the goals of captive breeding of APD in NA. In our study, microsatellites proved the most informative markers, followed by MHC, whereas D-loop provided the least information. Therefore, if time and funds are limited, we recommend that other institutions focus primarily on genotyping the same microsatellite markers, which would facilitate comparisons among studies of wild and captive populations. If additional data are required—for example, to help resolve uncertain parentage—sequencing of a nuclear locus could also be employed. Use of the DLA-DRB1 locus used here would facilitate comparisons among studies; however, other than finding that this locus is likely under balancing selection, it provided no further information about adaptability, for which it has purportedly been examined in APD [18] and other species [34,35,36,37,39]. Highly variable, noncoding nuclear loci such as introns may instead provide greater resolution for captive and inbred populations. Whichever markers are employed, future work with captive populations of APD should combine information for this species among regional captive populations across the globe with the goal of creating a genetically informed global management plan.

## Figures and Tables

**Table 1:** Relative frequencies of DLA-DRB1 haplotypes identified in the NA population of APD (this study) compared to European captive facilities and wild populations in southern Africa.

Allele Name	DRB Haplotype	NA (*n =* 104)	Europe (*n =* 211) ^a^	Wild (*n =* 185) ^b^
DRB*90101	A1	2.88	32.2	5.8
DRB*90102	A2	3.37	12.1	7.1
DRB*90201	A3	15.87	8.8	11.4
DRB*90202	A4	12.98	8.3	13.8
DRB*90301	A7	-	3.3	0.5
DRB*90401	B1	-	11.6	-
DRB*90402	B2	6.73	1.4	9.5
DRB*90501	B3	-	-	0.3
DRB*90601	B4	4.33	-	5.8
DRB*90602	B5	1.44	6.2	6.1
DRB*907011	B6	42.79	5.2	22.5
DRB*90801	B7	5.29	0.7	5.6
DRB*91001	B9	-	-	1.9
DRB*90901	B11	4.33	0.9	9.8
DRB*90702	B13	-	9.2	-

^a^ Data from [18].^b^ Data from [30,32].

**Table 2:** Indices of genetic diversity (*±* standard deviation, SD) based on 14 microsatellites amplified in the NA population of APD (this study) and compared to the European captive population [18], which was genotyped at 10 of the 14 microsatellite loci used in the current study. *n*, sample size; *N_A_*, mean number of alleles per locus; *R_S_*, allelic richness; *H_O_*, mean observed heterozygosity; *H_E_*, mean unbiased expected heterozygosity; *F_IS_*, inbreeding coefficient.

	North America	Europe ^a^
*n*	109	212
N_A_ ± SD (range of # alleles across loci)	6.750 ± 2.824 (4–14)	6.4
R_S_ ± SD	6.366 ± 2.598	4.8
H_O_ ± SD	0.816 ± 0.091	0.76
H_E_ ± SD	0.746 ± 0.086	0.73
F_IS_ ± SD	−0.108 ± 0.141	−0.05

^a^ Data from [18].

**Table 3:** Estimates of contemporary effective population size (*N*_e_) based on 14 microsatellites amplified in the NA population of APD—(this study), and compared to the European captive population [18], which was genotyped at 9 of the 14 microsatellite loci used in the current study. *N*_e_ was estimated in both populations using the linkage disequilibrium method [68], with a 95% confidence interval obtained through nonparametric jackknifing [71].

	*n*	*N_e_*	95% Confidence Interval	Assumed Mating System
NA	109	17.7	14.7–21.3	Random
NA	109	37.1	31.2–44.1	Monogamous
Europe	214	23.9	20.1–28.3	Random
Europe	214	49.5	42.0–58.2	Monogamous

## Data Availability

D-loop and MHC sequences have been submitted to GenBank (accession numbers MZ825436-MZ825437 and MZ825438-MZ825447, respectively), and microsatellite genotypes have been submitted to Dryad (https://doi.org/10.5061/dryad.xksn02vgp).

## Supplementary Materials

The following are available online at www.mdpi.com/article/10.3390/genes12101463/s1, Figure S1: Pedigree of a subset of the North American African painted dog population, including sampled individuals (*n =* 109), their parents, grandparents, and offspring. Squares represent males, ovals represent females. Colors indicate mitochondrial DNA haplotypes of sampled individuals (blue = S2, red = Z1) and illustrate that all but one individual with the Z1 haplotype are descended from a single wild-caught female.
