# Supplementary material for: Genetic Diversity and Relatedness among Captive African Painted Dogs in North America
Source: Genes (Basel). 2021 Sep 22;12(10):1463. doi: 10.3390/genes12101463 (PMC8535225; doi:10.3390/genes12101463)
Supplement: Supplementary file 1 [file genes-12-01463-s001.zip › genes-1366598-supplementary.pdf]

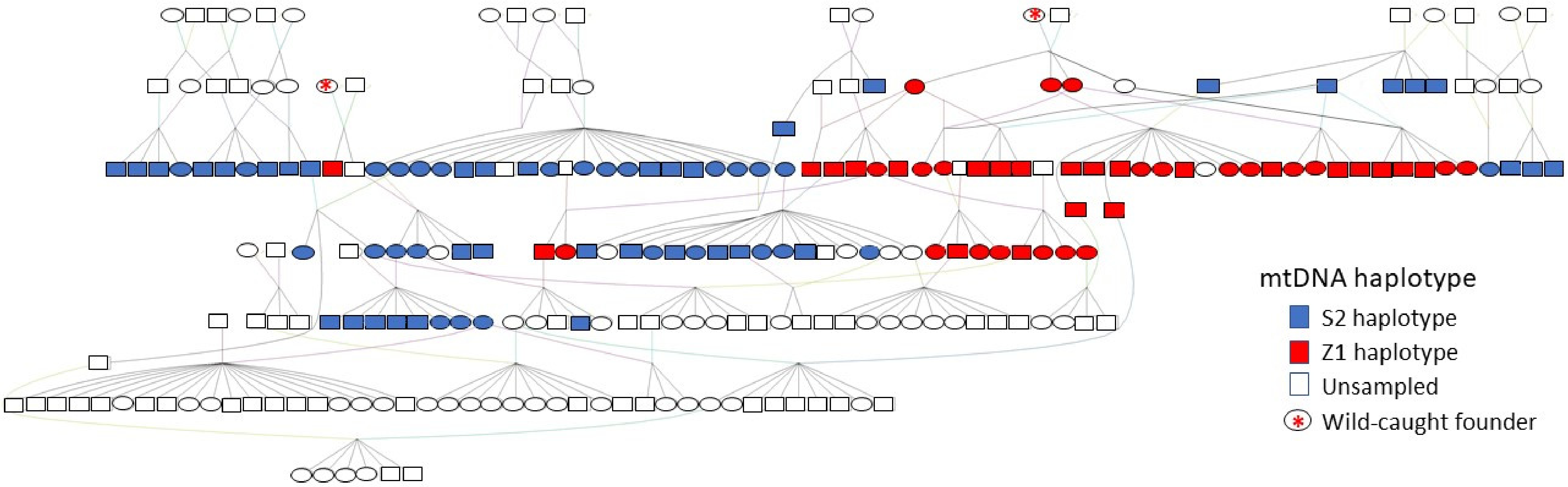

Figure S1. Pedigree of a subset of the North American African painted dog population, including sampled individuals (n = 109), their parents, grandparents and offspring. Squares represent males, ovals represent females. Colors indicate mitochondrial DNA haplotypes of sampled individuals (blue = S2, red = Z1) and illustrate that all but one individual with the Z1 haplotype are descended from a single wild-caught female.
